# Nano-immunotherapy: overcoming delivery challenge of immune checkpoint therapy

**DOI:** 10.1186/s12951-023-02083-y

**Published:** 2023-09-21

**Authors:** Seyed Hossein Kiaie, Hossein Salehi-Shadkami, Mohammad Javad Sanaei, Marzieh Azizi, Mahdieh Shokrollahi Barough, Mohammad Sadegh Nasr, Mohammad Sheibani

**Affiliations:** 1 Department of Formulation Development, ReNAP Therapeutics, Tehran, Iran; 2 Nano Drug Delivery Research Center, Health Technology Institute, Kermanshah University of Medical Sciences, Kermanshah, Iran; 3 Department of Medical Science, Tehran University of Medical Sciences, Tehran, Iran; 4 Cellular and Molecular Research Center, Basic Health Sciences Institute, Shahrekord University of Medical Sciences, Shahrekord, 8815713471 Iran; 5 Institute of Biochemistry and Biophysics (IBB), University of Tehran, Tehran, Iran; 6 Department of Immunology, School of Medicine, Iran University of Medical Sciences, Tehran, 1449614535 Iran; 7 Department of Computer Science and Engineering Multi-Interprofessional Center for Health Informatics (MICHI), The University of Texas at Arlington, Arlington, TX USA; 8 Department of Pharmacology, School of Medicine, Iran University of Medical Sciences, Tehran, Iran; 9 Razi Drug Research Center, School of Medicine, Iran University of Medical Sciences, Tehran, Iran

**Keywords:** Immune checkpoint, Nanoparticles, Chemoimmunotherapy, PD-1 and PD-L1, CTLA-4, Tumor microenvironment

## Abstract

Immune checkpoint (ICP) molecules expressed on tumor cells can suppress immune responses against tumors. ICP therapy promotes anti-tumor immune responses by targeting inhibitory and stimulatory pathways of immune cells like T cells and dendritic cells (DC). The investigation into the combination therapies through novel immune checkpoint inhibitors (ICIs) has been limited due to immune-related adverse events (irAEs), low response rate, and lack of optimal strategy for combinatorial cancer immunotherapy (IMT). Nanoparticles (NPs) have emerged as powerful tools to promote multidisciplinary cooperation. The feasibility and efficacy of targeted delivery of ICIs using NPs overcome the primary barrier, improve therapeutic efficacy, and provide a rationale for more clinical investigations. Likewise, NPs can conjugate or encapsulate ICIs, including antibodies, RNAs, and small molecule inhibitors. Therefore, combining the drug delivery system (DDS) with ICP therapy could provide a profitable immunotherapeutic strategy for cancer treatment. This article reviews the significant NPs with controlled DDS using current data from clinical and pre-clinical trials on mono- and combination IMT to overcome ICP therapeutic limitations.

**Graphical Abstract:**

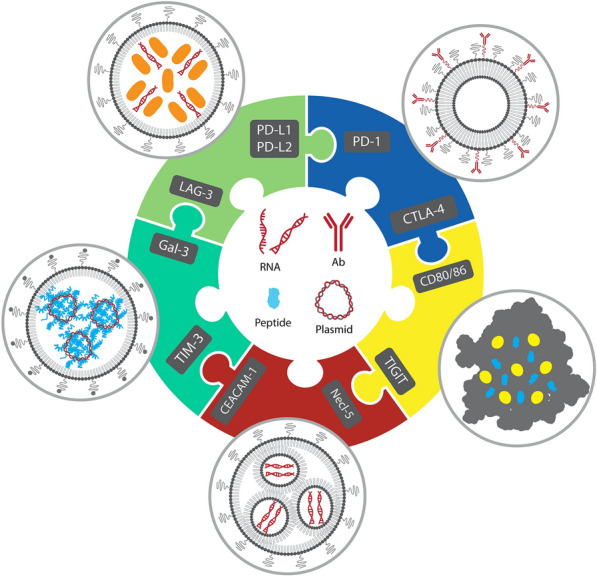

## Introduction

**Fig. 1:**
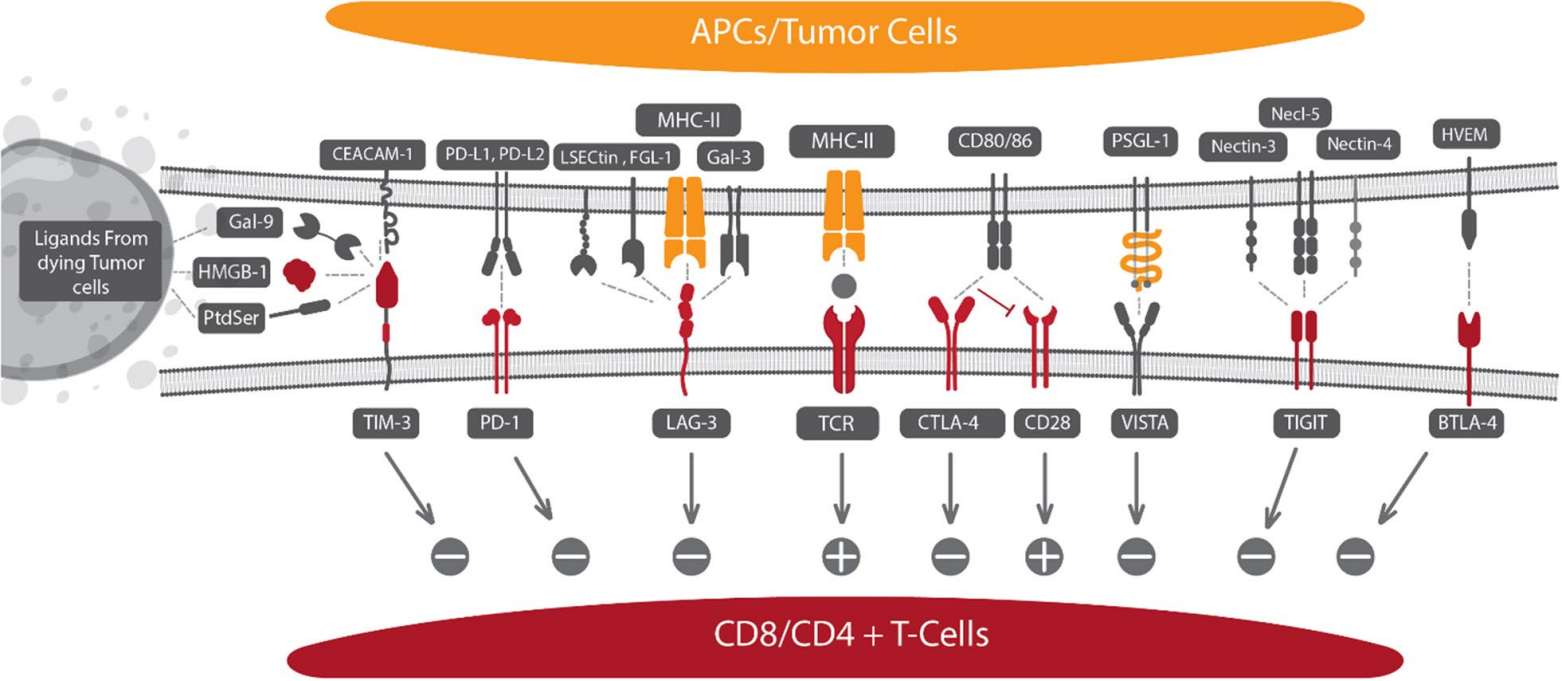
Representative profile of potential ICPs with relevant specific ligands and their function on CD8/CD4^+^ T cells. The interaction among negative co-inhibitory ICPs on CD8/CD4^+^ T cells, including TIM-3, PD-1, LAG-3, CTLA-4, VISTA, TIGIT, and BTLA-4 and positive co-inhibitory ICPs interact with TCR, and CD28 with their membrane protein of APC or tumor cells

The immune system is the most powerful arm in the body defense system to fight against tumors [1]. However, the tumor ability to escape this strong response makes cancer a progressive and hard-to-treat disease [2, 3]. Cancer immunotherapy (IMT) that focuses on immunoregulatory factors brings the cancer therapeutic method to another spirit [4–6] and includes the antibody(Ab) and cell therapy-based approaches currently under close investigation worldwide [7, 8]. Immune checkpoints (ICPs) are a variety of inhibitory mechanisms that are integrated into the immune system. They are essential for self-tolerance and regulating the latency and intensity of physiological immune responses in peripheral tissues to reduce collateral tissue damage. Tumors can control various immune checkpoint pathways as a primary immune resistance mechanism [9]. Novel ICP receptors, which include programmed death 1 (PD-1) and its ligand (PD-L1), and cytotoxic T-lymphocyte-associated antigen 4 (CTLA-4), are shown to suppress T cells presented at the tumor site [10, 11]. ICP inhibitors (ICI) are developed using antibodies (Abs), RNAs, peptides, or small molecules which can block ICP proteins. By the end of 2022, at least seven types of ICIs, including PD-1 inhibitors (Nivolumab, Cemiplimab, Pembrolizumab), PD-L1 inhibitors (Avelumab, Durvalumab, and Atezolizumab) and CTLA-4 inhibitor (Ipilimumab) have been approved by food and drug administration (FDA) for the various cancer therapies [12]. Furthermore, T cell immunoglobulin and mucin-domain containing-3 (TIM-3) [13], Lymphocyte activation gene-3 (LAG-3) [14], T cell surface protein containing an immunoglobulin variable (IgV) domain, a transmembrane domain and an immunoreceptor tyrosine-based inhibitory motif (ITIM), which is called TIGIT (T cell immunoglobulin and ITIM domain) [15], B and T lymphocyte attenuator (BTLA) [16], V-domain immunoglobulin suppressor of T cell activation (VISTA) [17], and B7 homolog 3 protein (B7-H3) [18] are next-generation of ICPs in the tumor microenvironment (TME). The schematic representative interplay of current ICPs with relevant specific ligands and their function on CD8/CD4^+^ T cells is shown in Fig. 1.

ICIs can activate systemic immune responses, leading to toxicities and resistance. Recently, ICIs indicate gained considerable attention in cancer therapy due to their exceptional significance in antitumor responses and long-term remissions [19–21]. It is no exaggeration to say that ICIs are among the most widely successful immunomodulators developed so far [22, 23]. However, ICIs have some kinds of disadvantages, like inducing numerous-immune related adverse events (irAEs) [24–26], disruption of the balance or regulation of immune responses [9], self-tolerance, and normal homeostasis of the immune system [27–29]. Thus, ICI therapy can cause myocarditis, autoimmune colitis, vitiligo, psoriasiform dermatitis, hepatitis, neuritis, and endocrinopathies such as type 1 diabetes and pancreatitis [26].

ICIs have limitations that combination therapy is adopted due to their adverse effcts including ICI side effects on human body, irAEs effect on clinical outcomes and ICIs endocrine side effects. In human body issue, ICIs work by releasing the brakes on the immune system, allowing it to attack cancer cells. However, this can also cause the immune system to attack normal cells in the body, leading to irAEs that can affect various organs and tissues.The most important irAEs of ICI therapy is as follows: Skin rash and Pruritus, Vitiligo, Colitis, Hepatitis, Pancreatitis, Myocarditis, Nephritis, Pneumonitis, Hypophysitis, Thyroiditis, Adrenal insufficiency, Type 1 diabetes and Neurological disorders (such as encephalitis, myasthenia gravis, and Guillain–Barre syndrome) [30]. Furethermore in a irAEs effect on clinical outcomes, the relationship between irAEs and clinical outcomes in all solid malignancies treated with ICIs was examined. A systematic review of the literature was conducted, and it was found that the development of irAEs was associated with better objective response rate (ORR), progression-free survival (PFS), and overall survival (OS) in patients with metastatic melanoma, lung cancer, renal cell carcinoma, urothelial cancer, head and neck cancer, and gastrointestinal cancers. It was also noted that grade 3 or 4 irAEs were associated with increased ORR but worse OS. The incidence of irAEs can be considered a predictive biomarker of treatment efficacy and toxicity associated with the use of ICIs, according to the study [31].

Despite the fact that there are many reports of endocrine side effects associated with cancer IMT, it is still not clear what their exact prevalence and mechanism. These adverse events include hypophysitis, thyroid disease, and primary adrenal insufficiency. Hypophysitis is a distinctive side effect of CTLA-4 blocking Abs, and prolonged or lifelong substitutive hormonal treatment is often required. The mechanism of injury to the endocrine system triggered by these drugs is yet to be fully elucidated, and well-designed studies are needed to find and validate predictive factors of autoimmune toxicity [32]. The irAEs associated with ICIs, can be distinct from conventional chemotherapy-related toxicities; this highlights the importance of awareness of the clinical presentation, diagnosis, and management of irAEs. The frequency of irAEs is dependent on the agents used, exposure time, and administered dose but also on the patient's intrinsic risk factors.

In order to reduce the side effects of ICI therapy and increase its therapeutic efficacy, it is essential to develop a drug delivery system (DDS) [33, 34]. To that aim, developing DDS and manipulating nano-biomaterial can help us design smart nano-carriers and overcome this barrier by directing ICIs toward our desirable location, achieving TME remodeling, and boosting anti-tumor immunity, and, subsequently, a safe and efficient cancer IMT [35–39].

Nanoparticles (NPs) emerged as significant tools by providing a targeted approach to effectively delivering cancer drugs. Inherent small size, shape, and flexible preparation of NPs, as well as numerous benefits like improved intracellular infiltration, hydrophobic solubility, reduced nonspecific uptake, and reduced toxicity of cancer therapy, all contribute to their ability to increase the efficacy and overcome the limitations of ICI therapy. Taken together, the superiorities of the combinational approaches with NPs and ICI originated from their ability to carry multiple cargos, protecting from nuclease, controlled release, diminished systemic harmful side effects, and modified pharmacodynamics (PD) effects of the cargos [40, 41]. The key idea is the use of confident vehicles to direct medications toward specific organs and cell types rather than systemic delivery, which is responsible for various off-target consequences. Likewise, the success of various nano-biomaterials as a tremendous carrier of ICI therapy was accurately demonstrated. In addition, it was shown that mono-immunotherapy (mono-IMT) with ICIs leads to higher tumor resistance and limited responses. Mono-IMT is the use of one ICI, which is drug that helps to activate the immune system to attack cancer cells. These drugs target specific proteins on the surface of cancer cells and immune cells, allowing the immune system to better recognize and attack cancer cells. Nano-immunotherapy (nano-IMT) has the potential to significantly improve the effectiveness of immunotherapy (IMT) treatments for cancer and other diseases. Thus, nano-IMT emerged to target different inhibitory factors or simultaneously impact both inhibitory and stimulatory pathways with an efficient delivery system [42–44]. Recent studies have shown that standard chemotherapy can improve the immune response to tumors and overcome immunoresistance in the TME. This has led to the idea of combining ICIs with standard chemotherapy as a way to enhance the effectiveness of cancer treatment [45, 46]. This novel approach not only leads to the efficient delivery of ICI with optimized dosage and perfect treatment within the body but also synergizes combinatorial therapy of ICI and other drugs (immune molecules and cells or chemotherapy drugs) due to dominating tumor immune evasion through reducing immunomodulatory agents’ exposure, and development of the unique combination of immunotherapies and improving the targeting efficiency treatment with manipulated and targeted NPs [47, 48]. The current review concentrated on the recent interaction between the NP-based delivery of ICI and its effects on cancer IMT. Indeed, we focused on the investigation that conducted the examination based on NPs and ICI simultaneously. Also, as one of this review aims, the combination of NP-based ICI and other inhibitory or stimulatory factors was discussed.

## Inhibitor agents for ICP pathways

### Antibody blocking agents

The cell–cell attachment and intracellular signaling cascade would charge T cells’ immunosuppressive features due to FOXP3 overexpression and IL-10, TGF-β production, which are crucial mediators of regulatory molecules in the immune system [2]. The ICP blocking modalities can neutralize the cell–cell attachment and induce apoptosis in ICP-expressing cells. The blocking monoclonal antibodies (mAbs) were the first line of ICP blocking systems. The FDA has approved three significant mAbs, including Nivolumab (Opdivo), Pembrolizumab (Keytruda), and Cemiplimab (Libtayo), against their ICP markers (Table 1) [3, 49]. These drugs can target the PD-1^+^ T cells and induce apoptosis in these cells. PD-1^+^ T cell depletion positively correlates with a good prognosis of cancer. The anti-PD-1 (aPD-1) treatment should be followed precisely to prevent the autoimmunity problems induced after continuous administration of the aPD-1 regimen. There are many aPD-1 agents in clinical trials, such as IgG1, ScFV, IgG4, single peptide, and some NP-conjugated formulations. New formulations of aPD-1 improve tumor infiltration of these Abs. The poly lactide-co-glycolide acid (PLGA)-loaded aPD-1 NPs can impact CD40^+^ and CD11c populations in mice tumor models, impacting dendritic cells (DC) activation, intratumoral interferon-γ (IFN-γ) production, and the tumor burden decrement [6]. Although products related to aPD-1 are expanding, the side effects of continued use of these ICIs have led to a greater focus on the design of treatment regimens based on PD-L1. The essential mAbs targeting PD-L1 known so far are Atezolizumab, Avelumab, and Durvalumab, which have received FDA approval. Finally, in 2020, Nivolumab plus Ipilimumab combined with platinum-based chemotherapy (two cycles) was approved by FDA as first-line treatment for metastatic or recurrent non-small cell lung cancer (NSCLC) [12]. Furthermore, the ongoing marketing products and clinical trials of ICI for aPD-1, antiPDL1(aPD-L1) and antiCTLA-4 (aCTLA-4) mAbs are listed in Table 1.

**Table 1:** FDA-approved ICI mAbs on the market

ICI types	mAb	Disease	Refs
aPD-1	Pembrolizumab	NSCLC^1^, HNSCC^2^, HCC^3^, RCC^4^, SCLC^5^, Melanoma	[49]
	Nivolumab	CRC^6^, HNSCC, SCLC, HCC, cHL^7^, RCC, NSCLC	[6]
	Cemiplimab	cSCC^8^	[12]
	Atezolizumab	MCC^9^, NSCLC, Urothelial carcinoma	
	Avelumab	Gastric cancer	
	Durvalumab	Urothelial carcinoma, NSCLC	
aCTLA-4	Ipilimumab	CRC, RCC, Melanoma	
aLAG-3, aPD-1	Relatlimab, Nivolumab	Melanoma	[50]

1. Colorectal cancer, 2. Head and neck squamous cell carcinomas, 3. Hepatocellular carcinoma, 4. Renal cell carcinoma, 5. Small-cell lung carcinoma, 6. Colorectal cancer, 7. Classic hodgkin lymphoma 8. Cutaneous squamous cell carcinoma, 9. Merkel cell carcinoma

### Nucleic acid-based blocking agents

ICP silencing via small interfering RNA (siRNA) or other inhibitory miRNAs can alter the expression level of ICPs and reduce the downstream signaling cascade protein function. Some NPs can enhance siRNA delivery into the tumor region and tumor-infiltrating lymphocytes (TILs). Some non-viral vectors, such as lipid-coated calcium phosphate (LCP) NPs, can improve the efficacy of siRNA entrance into TILs [51]. The oligonucleotide carriers should have some properties such as small size, cationic charge, lipophilic or amphipathic tendency, stability, clathrin-based endocytic capacity, and non-immunogenic phenotype. The cationic and polymeric NPs can achieve these properties, but the challenges encountered in manipulating, optimizing, and customized decoration for efficient and targeted delivery.

Furthermore, inhibiting ICPs with siRNA-containing NPs can improve the efficacy of cancer vaccines. In one study, combination therapy based on PD-1 and LAG-3 gene suppression in combination with DC vaccination was found to be a practical approach to breast cancer treatment. However, further studies need to be done [52]. The conjugated NPs for delivery of anti-ICP siRNA, such as PD-1, PD-L1, CTLA-4, and LAG-3 in cancer IMT, were shown in Table 2.

**Table 2:** The various ICIs and NDDS for cancer IMT using monotherapy and combinatorial therapies

ICI type	Target Disease	Nanoparticle type	Combination	Refs
PD-1, PD-L1 pathway inhibition				
PD-L1 siRNA	Breast cancer	N,N,N-Trimethyl chitosan (TMC)	–	[94]
		IR792-MCN^1^@ZIF^2^-8-PD-L1 siRNA (IM@ZP) NPs	–	[95]
		siPD-L1@PM^3^/DOX^4^/LP^5^ NPs	Doxorubicin	[96]
		PLGA^6^-based polymeric NPs	–	[97]
		Liposome	Birinapant	[98]
		Dextran NPs	–	[99]
		Cancer cell membrane-coated NPs (CCMNPs)	Doxorubicin	[100]
	Melanoma	(PEI^7^-PD-L1 siRNA complex) in liposome	Imatinib	[101]
		LNP^8^	–	[101]
		PDPA^9^	PDT	[102]
		PEG^10^-CDM^11^-PDEA^12^ and PEI-PDEA		[103]
	Melanoma and breast cancer	HA^13^-TAT^14^-TMC	STAT3 siRNA	[104]
	Lung cancer	cRGD^15^-targeted liposome	Anemoside B4 (AB4)	[105]
		PEI-LNPs	IL-2 DNA plasmid	[106]
	Gastric Cancer	FA^16^-PEG-PEI	–	[107]
	HCC	(TT-LDCP)^17^ NPs	IL-12 DNA plasmid	[108]
	Pancreatic cancer	Magnetic nanocarriers	–	[109]
Anti PD-1 Ab	Melanoma	pH-Sensitive calcium carbonate (CaCO_3_) NPs	Zebularine	[110]
		Microneedle composed of: HA-pH-sensitive Dextran@ aPD1 and Gox^18^	Glucose oxidase	[111]
		Inflammation-responsive nano-cocoons	TLR-9 agonist	[112]
		CaCO_3_ NPs	Zebularine (Zeb), an HMA	[110]
		pH Dual-Sensitive Micelles	Paclitaxel	[113]
	Melanoma and breast cancer	Maleimide-terminated PEG-PLGA	OX 40 agonist	[114]
	Breast cancer	DMSNs^19^@HA	anti-CD3 and anti-CD28 mimicking DCs	[115]
	Colorectal cancer	PEG-PLGA	TGFβ antagonist	[63]
Anti PD-L1 Ab	Colon cancer and melanoma	Iron-dextran NPs	4-1BB agonist	[116]
	Gastric cancer	Polyethylene glycol-poly(ε-caprolactone) NPs (PEG-PCL NPs)	Docetaxel	[5]
	NSCLC	ARAC construct	PLK1 inhibitor (volasertib)	[117]
	Glioblastoma	LNP	Dinaciclib	[118]
PD-L1 trap plasmid DNA	HCC	LCP^20^	–	[119]
	Pancreatic cancer	LPD^21^	CXCL12 antagonist	[120]
	Colon carcinoma		Oxaliplatin	[53]
Anti PD-1 peptides	Ovarian cancer	Cowpea mosaic virus (CPMV)	Photothermal therapy	[121]
	Breast cancer and CRC	PLGA and HAuNS^22^	–	[122]
Anti PD-1 mRNA	Intestinal cancer	LNP	Pembrolizumab	[123]
PD-L1 gRNA-CRISPR/Cas9 plasmid	Glioblastoma	PEI lipid (shell)-PLGA (core)	–	[54]
CTLA-4 pathway inhibition				
CTLA-4 siRNA	CRC and Breast cancer	Chitosan Lactate (CL)	–	[90]
	Melanoma	(PEG-PLA^23^), (BHEMChol^24^)	–	[124]
Anti CTLA-4 Ab	Melanoma	FMSN^25^	–	[125]
	CRC	pLHMGA^26^	–	[126]
Other pathways				
IDO inhibitor (NLG919)	Breast cancer	PSSN10^27^ prodrug polymer (Nano-micelles)	Doxorubicin	[127]
Tim-3 siRNA	HCC	(CC@SR&SF@PP)^28^	Sorafenib	[128]
Multi-pathway inhibition				
LAG-3/ PD-1 siRNA	Breast cancer	TMC-dextran sulfate—lactate	–	[52]
CD155 siRNA, PD-L1 Ab		mPEG^29^-PLGA-PLL^30^ (PEAL Nps)	–	[129]
PD-L1, IDO receptor	Melanoma	PD-1^+^ cell membrane-derived nanovesicles	–	[130]
PD-1, IDO Ab/1-MT^13^		HA	–	[131]
CTLA-4, PD-1 Ab	Glioblastoma	Poly (β-L-malic acid) based Nps	–	[132]
PD-1, PD-L1 siRNAs	CRC	PLGA based Nps	–	[133]
	Breast cancer	LCP	–	[51]

1.Mesoporous carbon nanocomposite; 2.Zeolitic imidazolate frameworks-8; 3.PAMAM dendrimer; 4.Doxorubicin; 5.Liposome; 6.Poly Lactic-co-Glycolic Acid; 7. Polyethyleneimine; 8. Lipid nanoparticles; 9. poly(2-(diisopropylamino)ethyl methacrylate); 10. Polyethylene glycol; 11. 2-propionic-3-methylmaleic anhydride; 12.Poly(2- (diethylamino) ethyl methacrylate; 13.Hyaluronic acid; 14. Transactivator of transcription peptide; 15.Cyclic arginine-glycine-aspartic acid; 16.Folic acid; 17.Tumor-targeted lipid-dendrimer-calcium-phosphate; 18.Glucose oxidase; 19. Dendritic mesoporous silica NPs; 20. Lipid-coated calcium-phosphate; 21. Lipid-protamine-DNA; 22. Hollow gold nano-shell; 23. polylactic acid; 24. *N-bis*(2-hydroxyethyl)-*N*-methyl-N-(2-cholesteryloxycarbonyl aminoethyl) ammonium bromide; 25.Functionalized mesoporous silica NPs; 26.Poly(D, L lactic-cohydroxymethylglycolic acid); 27.POEG(poly(oligo(ethylene glycol) methacrylate)) hydrophilic block and a NLG hydrophobic block; 28.Carboxymethyl chitosan(CMCS)@Tim-3 siRNA (SR) &Sorfeinb(SF)@mPEG5K-PAE10K (PP) NPs; 29.Methoxy-poly(ethylene glycol)-graft; 30.Poly(L-lysine)

Additionally, plasmid DNA can be used to localize PD-L1 trap protein expression along with siRNAs. Creating PD-L1 traps transiently and locally in the TME is possible by loading the PD-L1 coding plasmid DNA into lipid-protamine-DNA NPs, which can synergize with chemotherapy drugs to inhibit tumor growth [53]. Furthermore, PD-1/PD-L1 gene-editing tools are increasingly being investigated, but there are challenges to delivering these tools safely and effectively during clinical trials. As well they designed an NP delivery system using a low molecular weight PEI lipid coating and a PLGA core that can encapsulate a PD-L1 gRNA-CRISPR/Cas9 plasmid and transfect human U87 glioma cells expressing PD-L1 [54]. Using NPs to introduce a PD-L1 GFP-CRISPR/Cas9 plasmid into human glioma cells might provide a novel IMT platform to treat glioblastoma multiforme [54].

### Small molecule blocking agents

Targeted anticancer therapies are dominated by small molecules, while IMT uses antibody-based biologics. The widespread use of mAbs against cell surface markers has led to a greater understanding of immunoregulatory ligand-receptor pairs. [55]. mAbs indicate a significant advantage over small molecules in terms of their technical potential to generate selective drugs against biological targets. In addition to reducing off-target drug events, extensive knowledge of the immunoglobulin framework allows better performance of pharmacokinetic (PK) and pharmacology parameters [56]. However, mAb drugs face their limitations; mAb infusion regimens are less convenient in clinical practice than oral administration of small-molecule-based pills. More importantly, irAEs are more controllable in small molecules than Abs due to their shorter half-life and more effortless dose adjustment [55]. Even though ICI Abs have established themselves as the critical components in IMT, these potential advantages of small molecules over Ab drugs have ignited pharmacological efforts to interfere with the intracellular PD-L1-PD1 axis [55, 57]. Furthermore, increasing understanding of T cell intracellular signaling has revealed several negative feedback loops downstream of TCR engagement that could be targeted to boost antitumor T cell immunity. Mitogen-activated protein kinase1 (MAP4K1), also known as hematopoietic progenitor kinase 1 (HPK1) and diacylglycerol kinases (DGK) are two prominent examples. Negative feedback is also mediated by the tyrosine-protein phosphatase non-receptor type 6 (PTPN6, also known as SHP1) and PTPN22 enzymes, as well as the E3 ubiquitin-protein ligase CBL-B128 [55].

Small molecules have been thought to be incapable of inhibiting the PD-L1-PD1 interaction. Nonetheless, the first oral agents, such as CA-170 and GS-4224, have now entered clinical trials. CA-170 is derived from the amino acid sequence serine-asparagine-threonine, discovered through research using motifs from the PD1 primary sequence. According to the reports, the compound targets PD-L1 and VISTA [58, 59]. Even though small-molecule immuno-oncology drugs are becoming increasingly popular, studies on manipulating NPs to change their pharmacologic properties are lacking.

## Combination of ICI therapy with chemotherapy

Single-drug therapy has many disadvantages due to cancer heterogeneity and low efficacy. As a result, a combination therapy containing two or more therapeutic agents has developed. Chemoimmunotherapy (CIT) is a new phrase in immunology and oncology, referring to a new spectrum of combinatory cancer treatments. It is about chemotherapy and IMT [60]. Using mAb as an IMT approach besides chemotherapy can boost patient treatment responses. Therefore, ICI therapy, besides chemotherapies, would be included in some guidelines. Nowadays, drug-containing NPs conjugated with ICIs are one of the most critical approaches to CIT drugs. It is formulated by co-encapsulating the drug and ICI in a liposomal carrier or a cationic polymer for siRNA. It would be designed in the NPs-based DDS (NDDS) [61]. Paclitaxel and Carboplatin plus Ipilimumab have been used for NSCLC patients in phase II clinical trials [62]. Some predominant studies in combination therapy are held on NSCLC, such as KEYNOTE-189 (Pembrolizumab and other chemotherapy regimens like) [63], CheckMate 9-LA (Nivolumab and Ipilimumab) [64], and POSEIDON (Durvalumab) [65]. These studies were a combination of chemotherapy and IMT separately. The future of CIT will lead to NP-loaded drug-containing ICIs. aPD-1 and Cisplatin-NPs prepared in a microneedle can release the Cisplatin labeled with aPD-1. These NPs infiltrate the tumor site, and targeting T cells impacts tumor cells simultaneously [66]. Furthermore, the PD-L1 mAb decorated nano-liposome containing Paclitaxel induces more tumor regression than monotherapy [67]. The pH-sensitive nanomicelles containing paclitaxel and aPD-1 enhance immunogenic cell death while PD-1 blocks solid tumors [68].

In addition to Abs, siRNA can be used in CIT as another type of ICIs. Knocking out key genes involved in apoptotic processes and the cell cycle is one of the essential ways in cancer therapy. Hence, siRNA is a promising candidate for inhibiting tumor development and invasion [69]. Combining siRNA therapy with chemotherapeutic drugs can overcome multidrug resistance and promote apoptosis [70]. The presence of suitable DDS based on NPs is promising to overcome these challenges. Although concomitant delivery of ICI and pharmaceutical payloads with NPs are the most critical challenges of these DDS, these approaches indicate a promising feature of nano-IMT for ICI therapy compared to mono-line therapy approaches.

Recently, an innovative approach to vaccination and IMT using an implantable blood clot scaffold loaded with liposomes-protamine-hyaluronic acid NPs (LPH NPs) containing both a vaccine and siRNA. LPH-siRNA that targets PD-L1 and TIM-3 can reduce immunosuppressive signals in mature DCs and prevent the DCs from expressing a regulatory program in the scaffold. The scaffold is intended to recruit immune cells, particularly DCs, to create a DC-rich environment and enhance the immune response [71].

## Incorporation of NPs in ICI therapy

### ICI-NPs for tumor therapy

Compared with usual immunotherapeutic methods, ICI therapy notably exhibited numerous advantages. However, the major problem is systemic adverse events that could induce severe or life-threatening problems in some patients. Diarrhea, colitis, and flu-like symptoms are those adverse effects that could be managed, but endocrine-related effects and so on could not. Nanomedicine can help us overcome these shortcomings by providing novel and safe DDS [35]. To that end, the diversity of NPs gives them the ability to carry several therapeutic cargos, such as degradable agents and simultaneous soluble and insoluble drugs [72]. Likewise, we can use higher tolerated drug doses using NDDS due to their controlled release ability and fewer off-target effects [73].

Furthermore, NPs can target the local immune microenvironment instead of systemic impact, which makes the therapeutic method safer [35, 74]. Also, investigations revealed that NPs could penetrate the TME rather than conventional therapies by enhanced permeation and retention effect (EPR), thus accumulating in the TME [75]. Regarding the small size of NPs, they could pass through loose, tight junctions of tumor neo-vessels [76].

**Fig. 2:**
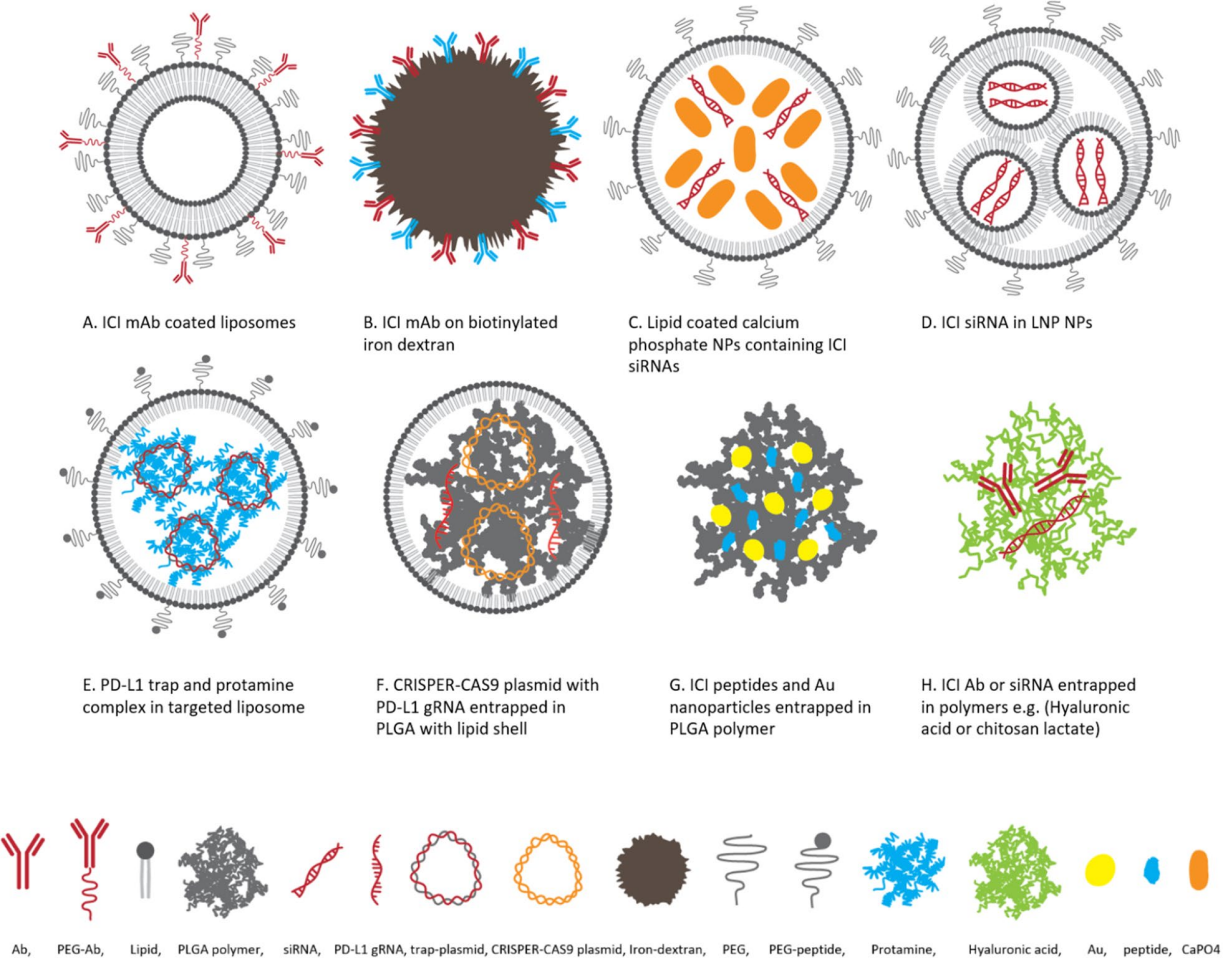
Schematic structure of NPs for delivering ICI Ab, siRNA, plasmid DNA, and peptides for cancer IMT

In the same way, the potential application of the NDDS can help us diminish toxic irAEs of IMT by the EPR effects and improve the risks of IMT by targeted therapy and systemic exposure reduction [76–78]. NDDS allows local delivery of ICIs without off-target exposure, thereby preventing auto-reactive and systemic immune responses. The manner, immunogenicity, and bio-distribution of NPs could be influenced by the size, shape, surface charge, decorating ligand, and density [79]. Overall, studies revealed the NDDSs importance in delivering ICIs into tumor cells, reducing toxic side effects, and improving anti-tumor responses. Several nanomaterials have been used to deliver biopharmaceuticals or IMT agents [73, 80]. In addition to enhancing immune checkpoint therapy, Nanoparticles have also been used for the Management of Immune-Related Adverse Events by ICIs [81]. Approximately two dozen clinically approved therapeutic products have produced nanoparticulate systems such as lipid-based, polymeric, inorganic, and hybrid NPs [80, 82–84]. The schematic structure of NPs for delivering ICI containing Ab, nucleic acid, and peptide ICIs for IMT is shown in Fig. 2.

The lipid vehicles for siRNA are among the newest platforms in gene delivery, which could be decorated as a targeting element, such as Ab labeling. It would prevent off-target involvement in healthy non-related cells or organs. These are bio-conjugated or multifunctional NPs [84]. The cationic NPs such as 1,2-dioleoyl-sn-glycerol-3-phosphate (DOPA) can induce the endosomal escaping and siRNA delivery in the cytoplasm [85], especially DOPA-coated dendrimer-siRNA/pDNA lipoid NPs, and the similar combinations can boost the efficacy of siRNA [86].

Unlike liposomal vehicles, which carry drug cargo in the lipid hydrophobic shell and inner hydrophilic core for taking lipophilic and hydrophilic drugs, respectively [87], polymeric NPs (as micelles and conjugates) comprise a robust and polymer-filled core that is better adapted for use in water-insoluble drugs. Attaching therapeutic agents to water-soluble polymers via a covalent bond is another strategy that enhances the lifetime of systemic circulation of drugs and decreases their exposure to normal tissues [85, 86]. Likewise, poly(ε-caprolactone) (PCL), PLGA, polyethyleneimine (PEI), chitosan, poly amido ethylenimine (PAE), poly(phosphazenes); p(DMAEMA), and poly amino amine (PAA) used as synthetic nano-polymers in the delivery. As the most popular one, PEI is used in gene delivery and increment of oligonucleotide or plasmids stability in the complicated process [88, 89]. PLGA, as an FDA-approved biocompatible and biodegradable polymer, shows non-linear and dose-dependent PK and targeted biodistribution features [61]. The majority performance of PLGA NPs is based on uptaking by DC without any specific character recognition, which is used for antigens, vaccines, and other immunotherapeutic agent delivery [62]. Furthermore, PLGA NPs as the carrier could be more helpful in inhibiting the immune escape of the tumor cells, inducing an antitumor-immune response, and blocking the immune check pathways for T cell activation [63, 65]. Chitosan (CS), as a semi-synthetic polymer, is the most identified polymer-based NPs [66, 90].

Inorganic nanomaterials include mesoporous silica NPs (MSN), and iron oxide NPs, play immune signal delivery due to unique physical features. Despite their immunogenicity, these NPs can enhance immune response, showing immunostimulant and immunosuppressant properties in multiple modes [67, 68, 91]. Hybrid NPs have also recently demonstrated promise for IMT molecules by combining organic compounds (lipids or proteins) with polymers, combining the advantages of tailor-made materials with the following advantages: extended circulation in the bloodstream, high encapsulation with various therapeutic agents, minimal premature leakage, targeted therapy simultaneously, and controlled release kinetics [92, 93].

### ICI-NPs for pleural and peritoneal effusion

Recent studies have highlighted the potential of NPs as a means of delivering drugs to the peritoneal cavity, particularly for treating peritoneal carcinomatosis. NPs possess numerous advantageous properties as drug carriers, including increased drug retention, prolonged action duration, and controlled drug release, making them effective vehicles for a variety of drugs, including ICIs [134, 135].

In a recent study, researchers investigated the potential of nano-IMT to treat peritoneal carcinomatosis in a mouse model of ovarian cancer. To deliver anti-PD-L1 to the tumor microenvironment (TME) in the peritoneal cavity, the researchers utilized a NPs-based delivery system known as IPI549@HMP. By selectively targeting tumor-associated macrophages (TAMs) within the peritoneal cavity, which play a critical role in promoting tumor growth and suppressing the immune response, the nanoparticles transported the anti-PD-L1 Abs to the intended site. The study demonstrated that delivering anti-PD-L1 Abs to the TAMs in the peritoneal cavity via the NPs platforms resulted in enhanced anti-tumor activity and improved survival in the mouse model [2]. Although there have been significant advancements in utilizing NPs systems for IMT in the treatment of peritoneal carcinomatosis, it is crucial to gain a more comprehensive understanding of the impact of locoregional therapy on the physiological and immune systems of human hosts [136].

## NPs and ICI targeting CTLA-4

As discussed, CTLA-4 is the protein expressed in T cells that can regulate CD28 expression [137]. CTLA-4 binding with CD80 and CD86 on APCs can reduce CD28 expression on T cells and inhibit T cell activation (Fig. 1). Thus, blocking of CTLA-4 may augment T cell function and boost anti-tumoral immune response. Therefore, the ICP, like CTLA-4, could be blocked by regulator Abs or the other inhibitor ligands, which is the origin of the ICI idea [138]. In this regard, several clinical trials were conducted in ICIs. Ipilimumab was the first FDA-approved ICI that could target CTLA-4 in metastatic melanoma [139]. It was demonstrated that CTLA-4 blocking led to an improved immune response by up-regulation of CD4^+^ T cells and down-regulation of T_reg_s [140]. Similarly, ICI for PD-1 (Nivolumab and Pembrolizumab) and PD-L1 (Durvalumab and Atezolizumab) are now approved to fight several kinds of tumors [141–143].

Rahimian et al. designed an investigation to release aCTLA-4 and anti-CD40 Abs sustainably by utilizing a biodegradable poly (D,L lactic-co-hydroxymethyl glycolic acid (PLHMGA) [126, 144]. The study was designed to evaluate microparticles larger than 10 µm for macrophages taken through the local release. The large-size microparticle escaped from macrophage uptake and concluded that aCTLA-4 receiving microparticles were sustainably released in colon carcinoma of mice and exhibited therapeutic efficacy equal 40% survival rate. Subsequently, that treatment strategy successfully reduced local side effects and serum levels of Abs [126].

Systemic administration of ICIs showed much lower potency against brain tumors than other types.[145–147]. However, systemic immune system stimulation caused by the free administration of ICIs might improve glioma-bearing mice's survival [148, 149]. To solve the problem and strengthen IMT, the drug should cross the blood–brain barrier (BBB) and penetrate the tumor. In a study of glioblastoma multiforme, a severe aggressive primary brain tumor [150], poly (β-L-malic acid) (PMLA) is utilized as a carrier for aCTLA-4 and aPD-1 Abs to deliver therapy into tumor cells. It was demonstrated that PMLA-based ICIs could cross the BBB and lead to local immune responses and higher survival of glioblastoma-bearing mice. The local treatment could elevate T cells and macrophage activity and reduce CD4^+^ FoxP3^+^ T cells (T_reg_s) in the tumor. Also, the natural killer (NK) cell population and the production of IL-4, 5, 6, and 10 were increased after therapy. PMLA-based ICIs were distributed in the tumor area but not in other healthy brain sites and inhibiting CTLA-4 and PD-1 in the tumor site. It was shown that PMLA-based aCTLA-4 significantly improved the local immune system in the tumor area and elevated the survival of glioma-bearing mice compared with the free drug [132].

In a study of B16F10 melanoma-bearing mice, researchers used a poly(ethylene glycol)-block-poly(D,L-lactide) (PEG–PLA) and the cationic lipid N,N-bis(2-hydroxyethyl)-N-methyl-N-(2- cholesteryoxycarbonyl-aminoethyl) ammonium bromide (BHEM-Chol) as NPs and CTLA-4-specific siRNA (siCTLA-4) as the ICI. Results showed a significant reduction of CTLA-4 in the activated T cells by administering NP-siCTLA-4. Also, the NP-siCTLA-4 regime, compared with NP-based siRNA-negative control, could induce the stimulation, activation, and proliferation of CD8^+^ and CD4^+^T cells remarkably and reduce CD4^+^ FoxP3^+^ T cells. In addition, NP-siCTLA-4 had a considerable role in delaying tumor development and increasing mice survival time compared to NP-based siRNA-negative control [124].

It was shown that local delivery of aCTLA-4 Abs through Montanide ISA-51 as a slow-release mechanism in the mice-bearing tumor could provide immune responses (higher CD8^+^ T cells) tumor eradication in low dosages of the drug. Consequently, this low plasma level of ICI minimized the irAEs as the treatment didn't increase autoAbs levels [125, 151].

In another study, functionalized MSN (FMS) was used as an interactive nano environment, elevated protein activity, and carrier with high protein load [152]. It was demonstrated that aCTLA-4 is loaded in FMS with superhigh density to be released long-lastingly. FMS-aCTLA-4 improved the therapeutic responses in melanoma models considerably compared to the free drug released systematically. Interestingly, the rate and durability of aCTLA-4 could be modified through the changes in functional group types and coverages of FMS [125, 148, 152, 153].

In the colorectal cancer (CRC) study, multifunctional upconversion NPs were utilized. This platform was composed of a photosensitizer chlorin e6 (Ce6), and a toll-like receptor-7 (TLR-7) agonist (imiquimod (R837)) as the immune adjuvant, together with aCTLA-4 was able to target tumor growth effectively. The combination therapy potentially eradicates the primary tumors and plays a vital role in the distant tumors' hindrance. This is while unaccompanied UCNP-Ce6-R837 and/or aCTLA-4 could not eliminate the tumor and had only a partial tumor growth delay. The strategy also provoked memory immunity which could support any possible disease recurrence [82, 154].

Altogether, it was shown that CTLA-4 blockades in each way, e.g., mAb or siRNA with the kind of NPs, can improve the anti-tumor efficacy of therapeutic strategies. In addition, other therapeutic factors showed synergistic effects to boost cancer IMT in combination with CTLA-4 blockades. Besides, the NP-based approach makes CTLA-4 blockades penetrate the tumor site and augment the immune system locally, reducing irAEs.

## NPs and ICI targeting PD-1/PD-L1 and IDO

### ICI targeting PD-1/PD-L1 using NPs

PD-1, one of the inhibitory molecules expressed on the T cell surface, could bind to PD-L1, a surface molecule of cancer cells, and induce inhibitory function [155]. Typically, the interaction between PD-1 and PD-L1 is necessary to maintain immune homeostasis and tolerance [156, 157]. Despite the beneficial function of the PD-1 and PD-L1 axis in normal conditions, studies have focused on blocking this pathway by using ICIs to inhibit tumor growth and improve cancer IMT [21]. There was an association between PD-1/PD-L1 expression level and poor prognosis of the disease and cancer recurrence, especially in breast cancer [158, 159]. In addition, PD-1 expression increased when the disease turned to the late stage [158]. PD-1 protein expressed on lymphocytes, specifically T cells, binds to PD-L1. The PD-1-PD-L1 expression on the tumor cells leads to the tumor escape from T cell-mediated anti-tumoral responses [138].

In an orthotopic CRC model study, an engineered PD-L1 trap plasmid DNA in lipid-protamine-DNA (LPD) NPs was utilized as the alternative to systemic aPD-L1 mAb therapy. It was demonstrated that the combination therapy of the PD-L1 trap (via fusion) with Oxaliplatin showed an improved anti-tumor efficacy and reduced toxic side effects associated with systemic aPD-L1. This is while aPD-L1 mAb plus Oxaliplatin could induce considerable Th17 accumulation in the spleen compared to the NP-PD-L1 trap [53]. Similarly, in a study of liver metastasis of CRC, lipid-coated calcium-phosphate (LCP) NPs were applied as the carrier of PD-L1 and CXCL12 trap plasmids to deliver into the hepatocyte nucleus. The treatment was shown to remarkably elevate immunotherapeutic factors and reduce immunosuppressive agents' concentration in the liver, thus demonstrating higher efficacy compared with the free drug IMT [119]. LCP NP-based therapy was further studied because of its high biocompatibility, good biodegradability, and low toxicity [51, 160]. They also have excellent activity in endosome escape and release of siRNA [161]. The study was designed to knock down the PD-1 of tumor lymphocytes and PD-L1 of tumor cells or possibly APC through the siRNA system to boost tumor-specific lymphocyte responses in an ex vivo model. According to the results, it was revealed that simultaneous blocking of PD-1 and PD-L1 significantly augmented the breast tumor-specific T cell responses. Thus, it could be helpful in T cell-based therapy for cancer patients. Results also demonstrated the strategy elevation of pro-inflammatory cytokines (PICs) such as IFN-γ and tumor necrosis factor (TNF-α). Also, this NP-based ICI was shown as an effective method for delivering cargo into the TME. They claimed that siRNA-mediated therapy is better than the antibody option (Abs) with a short half-life in multiple treatments [161, 162]. An interesting study compared the efficacy of two different NPs, such as layered double hydroxide (LDH) and LCP, to carry for PD-1 specific siRNA (siPD-1). As data showed, LCP NPs had higher cellular uptake and more potential to silence the PD-1 gene in mouse T cell line EL4 than LDH. Besides, LCP NPs showed significantly reduced PD-1 expression in human ex vivo TIL [163].

A study of gastric cancer that utilized folic acid (FA)-modified PEG-S = S-PEI complexes accompanied by superparamagnetic iron oxide NPs for delivering a PD-L1-specific siRNA (si-PD-L1). FA can bind to folate receptor (FR), which is overexpressed in many cancers, including gastric cancer cells, and can boost the complexity of cellular uptake. The FA-PEG-PEI polymers successfully delivered si-PD-L1, with lower off-target toxicity and considerable cellular uptake. In addition, this regime resulted in PD-L1 downregulation in both mRNA and protein levels, further elevating the cytokine release of cocultured T cells [107].

A study of tumor growth used an NP-based dual-targeting therapeutic strategy for combination IMT. The NPs applied were termed immunoswitch particles because they could switch off the PD-L1 on tumor cells and switch on 4-1BB (a co-stimulatory factor) on CD8^+^ T cells. The conjugation of Abs and iron-Dex NPs led to a synergy between two IMT approaches and a safer way to be useful at low dosages. In vivo analysis of colon cancer and murine melanoma showed that the NP-based combination dual-targeting therapy had a significant antitumor function. The treatment elevated the number and specificity of anti-tumoral CD8^+^ T cells and changed the endogenous T cell receptor repertoire. In addition, they demonstrated a more impressive recognition ability to identify tumor antigens. However, the immunoswitch strategy hampered tumor growth significantly compared with soluble aPD-L1 and a4-1BB mAbs. This is while particles themselves no indicate any advantages despite the intratumoral injection. Indeed, the conjugation of Abs and NPs is a necessary option for therapeutic anti-tumor activity. It was also demonstrated that bioactive particles had higher local concentrations and lower off-target exposure [116].

The combination therapy concept was further continued with another model study of the B16-F10 melanoma cells and immune-primed 4T1 breast cancer cells. A dual-immunotherapy NPs (DINP), an aPD-1 antagonist, and an anti-OX40 (aOX40) agonist were used as combination therapy. DINP was make-up by conjugating aPD-1 and aOX40 to maleimide-terminated PEG-PLGA NP through thiol-maleimide chemistry. Data showed that the DINP system combination therapy could induce IMT more effectively than free antibody administration. 83% of cured mice eliminated the tumor recurrence, showing the combination therapy that prolonged anti-tumor immune memory responses. Moreover, DINP combination therapy elevated the frequency of CD8^+^ T cells, the ratio of CD8^+^ to T_reg_s, and the effector to central memory T cells compared with the free antibody-administered group. It was shown that synergistic IMT with DINP promoted T cells' activation compared with free antibody IMT [114].

A study of mice bearing subcutaneous B16F10 melanoma, aPD-1, and Zebularine (Zeb) was investigated as a combination therapy. pH-sensitive CaCO_3_ NPs received aPD-1 for local controlled release. This NP-based aPD-1 was encapsulated with Zeb into the ROS-responsive hydrogel (Zeb-NP-based aPD-1-Gel). As results showed, this NP-based treatment considerably inhibited the tumor compared with the blank-Gel group via the augmentation of T cell-mediated anti-tumor responses. Furthermore, Zeb-NP-based aPD-1-Gel therapy was concentrated accurately in the target site. Compared with the control group, it was shown to not have off-target toxic side effects in the heart, liver, spleen, lung, and spleen [110].

An investigation of the MC38 model of CRC, aPD-1, in conjugation with PEG-PLGA NPs co-encapsulated with TGFβ inhibitor (SD-208), significantly augmented survival rate compared with free drug. It was shown that this treatment reduced off-target toxic adverse effects as the treatment was released after reaching infiltrated T cells [63].

The aPD-1 and glucose oxidase (GOx) were encapsulated into a microneedle patch containing HA grafted with pH-sensitive Dex NPs to treat the B16F10 mouse melanoma model. This NP-based APD-1 treatment was released sustainably because of the acidic condition of the TME. As a result, the MN-GOx patch, which can deliver aPD-1 (MN-GOx-aPD1), demonstrated continuous tumor suppression. Notably, 40% of mice survived 40 days after the therapy, whereas no one survived in the control group. In addition, the infiltration of CD4^+^ and CD8^+^ T cells in the MN-GOx-aPD-1 treated group was considerably higher than the untreated one. Furthermore, the combination of aCTLA-4 and aPD-1, with the help of microneedle as a carrier, had a considerable synergistic improvement compared with free Abs. This combination therapy interestingly led to complete control of melanoma as it showed long-term disease-free survival in 70% of treated mice in 60 days [111].

Another study designed a modified platelet system as a biological carrier with the help of ICI in postsurgical cancer IMT. Based on intrinsic platelet properties and bifunctional maleimide linker assistance, platelets were conjugated with a-PD-L1 (P-aPD-L1) to control postsurgical tumor recurrence and metastasis. As platelets interact with circulating tumor cells, aPD-L1 could target tumor cells in the blood circulation and surgical sites. According to results, P-aPD-L1 treated mice showed a 75% survival rate in 60 days, while no mice of all groups survived more than 30 days. As a result, this treatment strategy remarkably diminished cancer growth and metastasis risk and elevated the postoperative survival rate [164].

An investigation in postoperative tumor relapse utilized inflammation-responsive nano-cocoons as the carrier of aPD-1 as the ICI, along with CpG oligodeoxynucleotides (CpG ODNs) as an immune stimulator [112]. CpG ODNs play their potent immunostimulatory function by triggering TLR9-containing cells, which could boost anti-tumor function [165, 166]. In the B16F10 mouse melanoma models, this NP-based ICI therapy, with the simultaneous help of immunostimulators and immunosuppressive inhibitors, hampered tumor relapse more impressively than free aPD-1 and/or CpG nucleotide treatment. It was also demonstrated that the CpG-based delivery of aPD-1 improved the therapeutic method after fragmentation [112].

In a preclinical allograft pancreatic cancer study [120], a liposome-protamine-DNA (LPD) NP-based therapy was designed to treat the disease with the help of plasmids encoding small trapping proteins targeting PD-L1 and CXCL12 (a major chemokine which could inhibit the T cells infiltration [167]. The therapy regulated the TME and boosted the T cell infiltration. Also, it was shown to diminish the metastasis of the tumor cells remarkably. This local and transient delivery of NP-PD-L1 and CXCL12 was shown to reduce irAEs compared with free drug administration [120, 168].

Another recent study used the NDDS to deliver ICIs into the tumor sites. IO@FuDex, composed of iron oxide NPs, fucoidan, and aldehyde-functionalized Dex, was linked with aPD-L1 as the ICI, and aCD3/aCD28 was used as an immune system activator. This new strategy improved the efficacy of the combination therapy in tumor-bearing mice (breast cancer, lung metastasis, and colon cancer) and reduced the drugs' systemic accumulation. As a result, the median survival of tumor-bearing mice increased from 32 days (free aPD-L1 trial) to 63 days [169].

The therapeutic effects of PDT and ICI as combinational therapy were investigated in another B16-F10 melanoma xenograft tumor model study. A combination of PDT through an acid-activatable versatile micelleplex was incorporated by a siPD-L1. The micelleplex utilized a pH-responsive diblock copolymer poly(ethylene glycol)-block poly(diisopropanol amino ethyl methacrylate-cohydroxyethyl methacrylate) (PEG-b-P(DPA-co-HEA) (PDPA), that grafted with a photosensitizer of Pheophorbide A (PPa), and 1,2-epoxytetradecane alkylated oligoethyleneimine (OEI-C14), which is necessary for siRNA complexation and delivery, and the siPD-L1 to silence the PD-L1 expression on the surface of tumor cell. The frequency of CD8^+^ TIL and the IFN-γ level was remarkably higher in the combination therapy group than in the control ones. The PDT and PD-L1 blockade therapy showed many anti-tumor responses as this regime eliminated tumors utterly. Also, the method was demonstrated to suppress lung metastasis, which is the event in the B16F10 tumor [102].

Similarly, in another study of the B16F10 melanoma tumor, researchers combined PDT therapy and ICI with a micelleplex-based pH-responsive nanocarrier. The therapeutic method focused on the blockade of PD-1/PD-L1 interaction by a siPD-L1 which enhanced anti-tumor immune response via mitochondria-targeting photosensitizer (MTPP) simultaneously. The micelleplexe was made up of two copolymers, including PEG block conjugated poly(2- (diethylamino) ethyl methacrylate (PDEA) (PEG-CDM-PDEA), linking with a pH-sensitive amide bond from 2-propionic- 3-methylmaleic anhydride (CDM) molecule and PEI conjugated PDEA copolymer (PEI-PDEA) which has a potent affinity to bind siRNA. The mild acidic TME cleaves the amide bonds, leading to PEG layer loss and PEI middle layer exposure. The next steps depend on the low pH of endo-lysosomes, which is the cause of disassembly and cargos release. The released siRNA could silence the PD-L1 gene and reduce immune resistance. This is while the laser irradiation through stacked-up MTPP in the mitochondria induces cell apoptosis. Consequently, the therapeutic strategy improved anti-tumor responses and inhibited melanoma metastasis in mice. Also, the primary treated mice's survival rate was 83% after 30 days, remarkably higher than other groups [103]. PD-L1 mAbs copolymerized with PEG-PCL NPs resulted in enhanced uptake of aPD-L1 by tumor sites of mice models [5]. In addition, The conjugated NP formulations of aPD-L1 based on all-trans retinoic acid (ATRA), PLGA, and PEG have more drug delivery potency and more efficacy [170]. Overall, PD-1/PD-L1 blockade was used to overcome problems associated with monotherapy with ICIs. Based on the results of this study, NP-based PD-1/PD-L1 blockade platforms increased IMT accuracy by a significant margin, reduced toxic off-target effects, and provided an extended immunological memory activity that may prevent tumor recurrence after initial eradication.

### ICI targeting ADO-PD/ADO using NPs

Research in the B16F10 melanoma model applied engineered cell membrane-derived nanovesicles which presented PD-1 to improve the cancer IMT. This method showed the ability to disrupt PD-1/PD-L1 interactions by binding to the surface PD-L1 of tumor cells. Furthermore, to increase the therapy’s effectiveness, 1-methyl-DL-tryptophan (1-MT), an indoleamine-2,3-dioxygenase (IDO) inhibitor, was encapsulated into PD-1nanovesicles (NVs) [130]. IDO is a rate-limited enzyme in the catabolism and degradation of tryptophan [171] which could inhibit T cells function [172]. Thus, the therapeutic method could simultaneously hamper the PD-1/PD-L1 pathway and IDO. Consequently, in vivo data showed that the therapeutic strategy boosted tumor-specific immune responses by reducing exhausted CD8^+^ T cells and improving their anti-tumor effects. The therapy significantly delayed the B16F10 tumor growth in the PD-1 NV-administered group compared with aPD-L1 antibody alone. The PD-1 NVs treatment caused 20% of the mice to survive more than 60 days. This is while NVs alone had no remarkable anti-tumor effects [130].

In a study of 4T1tumor-bearing mice, a new redox-sensitive system named PSSN10 consisted of a POEG hydrophilic block and a PNLG hydrophobic block with some NLG919 motifs was used to co-deliver NLG919 (a nontoxic IDO1-selective inhibitor) as an ICI in combination with DOX as a chemotherapy agent. The PSSN10 prodrug polymers that carry NLG919 showed a self-assembly ability to make nanoscale micelles for DOX loading. As a result, this system led to more combination therapy (NLG919 and DOX) accumulation in the tumor sites. Rapid release of DOX was observed when the carrier got to the tumor site. Beyond the chemotherapy effects of DOX, it could augment tumor antigen presentation and promote anti-tumor immune responses. This is while the covalent linkage of NLG919 and a polymer made the cargo release slower and enhanced immunity for an extended period of time. It could be concluded that DOX/PSSN10 mixed micelles showed a significant tumor-inhibitory effect and higher survival of treated mice than free drugs. This was due to the simultaneous release of NLG919 and DOX at the tumor site [127].

A recent study of B16F10 melanoma investigated the synergistic effect of MN-based transcutaneous delivery of aPD-1 and IDO blockade with 1-MT. This strategy was able to release the therapeutic factors in the TME sustainably locally. As a result, 70% of mice bearing melanoma survived 40 days after the therapy, whereas none survived in the related control group. Consequently, this NP-loaded microneedle considerably improved the therapeutic effects and reduced systemic exposure-related side effects [131].

## NPs and emerging ICIs

There has been an increase in interest in emerging ICIs and their combination with other approved IMT drugs in recent years. Recently anti LAG-3 Ab (Relatlimab) has gained FDA approval in combination with nivolumab for melanoma cancer treatment [50]. Here again, NPs can be used for targeted therapy and enhance PK parameters of ICI. One research used trimethyl chitosan-dextran sulfate-lactate (TMC-DS-L) NPs loaded with siRNA molecules to inhibit the PD-1 and LAG-3 expressions.

The inhibition of ICP receptors such as LAG-3 and PD-1 on T cells was investigated in this study in order to increase the efficiency of T cells in response to DC vaccines [52]. T cell immunoglobulin mucin-3 (Tim-3) is a newly discovered immune checkpoint molecule and a promising target for HCC treatment [14, 128]. Song et al. developed a novel pH-triggered drug-eluting NP (CC@SR&SF@PP) for simultaneous administration of Tim-3 siRNA and sorafenib to HCC in vivo. Following pH-triggered sorafenib release from SF@PP NPs, tumor proliferation and angiogenesis were significantly inhibited, resulting in remarkable tumor growth suppression in a mouse hepatoma 22 orthotopic tumor model. As a result, the co-delivery of Tim-3 siRNA and sorafenib via this unique pH-triggered drug-eluting NP improves the anti-tumor effect [128]. We anticipate that these combination treatment methods will have tremendous promise in future clinical uses.

In addition to the conventional use of NPs as a delivery system, they can be used in conjunction with IMT as phototherapy (PT) agents. PT approaches commonly use light stimulation to treat peripheral infections and solid tumors due to synergized therapeutic performance, low toxic side effects, and less pain in patients than chemotherapy and radiotherapy [173, 174]. Furthermore, the studies demonstrated that light stimulation could generate tumor-associated antigens (TAAs) and promote innate immune responses [138]. Likewise, photothermal therapy (PTT) had a function in the induction of immune responses and played an adjuvant role [139]. Despite the tremendous anti-tumor ability of PT there is a need to augment anti-tumor immune responses due to the numerous evasion mechanisms that tumors employ to dampen anti-tumor activities [105]. With this regard, the combination therapy of light-triggered PTT with ICI accompaniment was examined in the primary tumor ablation context. Single-walled carbon nanotubes (SWNTs) were combined with a aCTLA-4 antibody and showed an inhibitory role in developing mouse tumor metastasis. Moreover, the polymer-coated SWNTs not only had a photothermal function for the demolition of the tumor, which further led to the release of TAAs, but also demonstrated immunological adjuvant effects as it could induce the maturation of DCs. With the kinds of aCTLA-4, this combination therapy promoted the T cells infiltration into the tumor site. At the same time, it could override the activities of regulatory T cells in distant tumors. It was demonstrated that in a subcutaneous tumor model and a distant established lung metastasis model, SWNT-based PTT combined with aCTLA-4 hampered the growth of remaining cancer cells [105].

Some PTT besides NPs can synergize ICP inhibitory effects, such as Prussian blue NP (PBNP) conjugated anti-CTLA-4 (aCTLA-4) mAbs [140]. The NP-mediated hyperthermia via aCTLA-4 mAbs can cause to depletion of T_reg_s in tumor masses [141]. PDT is another PT therapy that applies singlet oxygen (^1^O_2_) or other reactive oxygen species (ROS), which are the products of photosensitizer molecules enhancement to a high-energy level by a proper excitation light and kills tumor cells [142, 143]. It was exhibited that small peptides are a promising treatment for the ICIs as they can compete with high ICI therapy costs based on Abs. However, the peptides were shown to have some disadvantages beyond the low cost, like short blood circulation time, serum level instability, and side effects [82, 175]. In a study of murine 4T1 breast cancer and CT26 CRC, PLGA NPs were used to load an APD-1 peptide into a hollow gold nanoshell that induced photothermal ablation (PTA) [122]. PTA is another PT method that utilizes the light-generated heat of a near-infrared laser as a weapon to damage tumor cells [176]. This treatment strategy revealed potent anti-tumor responses, which significantly suppress the growth of colorectal and breast cancer. Furthermore, the slow degradation of PLGA led to a controlled release of a aPD-1 peptide, which made this therapy more effective by concentrating on the TME and blocking PD-1 efficiently [122].

## Conclusion

Despite the acceptable number of sufferers from specific cancer types responding to ICI therapy, new combinatorial strategies synergized ICI therapy provide a cure or improve the quality of overall survival, at least due to overcoming primary and adaptive resistance performances. Nano-IMT facilitates therapeutic potency by enhanced sequential monotherapy through targeted site-specific or combination therapy to maximize efficacy and minimize toxicities. Thus, combining ICIs with chemotherapy or immunopharmacuetical agents provides flawless simultaneous delivery for T cell activation, immune cellular and molecular modulation and regulation, and assurance of clinical trials. Our review indicates the incorporation of NPs with ICI therapy postulates a promising way to go by applying lipid, polymeric or hybrid NPs for the antitumor treatment and inorganic and metallic NPs for theranostic and imaging purposes. Despite the increasing development of lipid-based nanoparticles (such as liposomes and LNPs) in clinical trials for delivering RNA and immunotherapeutic agents, their utilization for ICIs has been inadequate. However, lipid-based NPs hold promising potential as carriers for ICIs, particularly for antibody or RNA cargoes aimed at blocking ICP pathways.

In addition, polymer NPs act as pioneer immune-NPs due to the activation of APCs and T cells, promotion of effector cells, ease of preparation, quick manipulation, effective targeting, and FDA approval of polymer NPs expanded use for ICI therapy in laboratory research and clinical trials. Furthermore, recent advances demonstrate that the hybrid NPs (lipid-polymer, polymer–polymer, and inorganic-polymer are the perspectives of combination IMT. Likewise, the next decade of ICI therapy will be established using the development of engineered carriers through targeted NPs delivery and RNA and Ab payloads for solid tumors.

Finally, various combination therapies have been developed using mAbs and other therapeutic agents to enhance the efficacy of ICT. Likewise, combining different mAbs that target different ICPs can lead to a more comprehensive blockade of ICPs, resulting in a stronger anti-tumor immune response. Additionally, combining mAbs with chemotherapy or other targeted therapies can help to overcome tumor resistance and enhance the effectiveness of the treatment. Another approach is the use of siRNA to target specific genes involved in cancer growth and survival which used in combination with monoclonal antibodies or other therapies to further enhance the anti-tumor response. Overall, the development of combination therapies using mAbs and other agents has significantly improved the efficacy of immune checkpoint therapy, and has led to the development of new treatment strategies for a wide range of cancers. Ongoing researches which discussed are focused on identifying the most effective combinations of therapies and optimizing treatment protocols to maximize patient outcomes.

## Data Availability

Not Applicable.

## Abbreviations

TCR: T-cell receptor
MHC-II: Major histocompatibility class II
PD-1: Anti-programmed death-1
CTLA-4: Cytotoxic T-lymphocyte-associated protein 4
LAG-3: Lymphocyte‐associated gene 3
CD: Cluster of differentiation
VISTA9: V-domain Ig suppressor of T cell activation
BTLA-4: B and T lymphocyte attenuator 4
GAL-9: Galectin-9
HMGB-1: High mobility group box 1
PtdSer: Phosphatidylserine
CEACAM-1: Carcinoembryonic antigen-related cell adhesion molecule 1
LSECtin: Liver and lymph node sinusoidal endothelial cell C-type lectin
PSGL-1: P-selectin glycoprotein ligand-1
HVEM: Herpes virus entry mediator
Necl-5: Nectin-like molecule-5
FGL-1: Fibrinogen-like protein 1
