# Correction: Nano-immunotherapy: overcoming delivery challenge of immune checkpoint therapy

**DOI:** 10.1186/s12951-023-02199-1

**Published:** 2023-11-18

**Authors:** Seyed Hossein Kiaie, Hossein Salehi‑Shadkami, Mohammad Javad Sanaei, Marzieh Azizi, Mahdieh Shokrollahi Barough, Mohammad Sadegh Nasr, Mohammad Sheibani

**Affiliations:** 1 Department of Formulation Development, ReNAP Therapeutics, Tehran, Iran; 2 Nano Drug Delivery Research Center, Health Technology Institute, Kermanshah University of Medical Sciences, Kermanshah, Iran; 3 Department of Medical Science, Tehran University of Medical Sciences, Tehran, Iran; 4 Cellular and Molecular Research Center, Basic Health Sciences Institute, Shahrekord University of Medical Sciences, Shahrekord, 8815713471 Iran; 5 Institute of Biochemistry and Biophysics (IBB), University of Tehran, Tehran, Iran; 6 Department of Immunology, School of Medicine, Iran University of Medical Sciences, Tehran, 1449614535 Iran; 7 Department of Computer Science and Engineering Multi-Interprofessional Center for Health Informatics (MICHI), The University of Texas at Arlington, Arlington, TX USA; 8 Department of Pharmacology, School of Medicine, Iran University of Medical Sciences, Tehran, Iran; 9 Razi Drug Research Center, School of Medicine, Iran University of Medical Sciences, Tehran, Iran

**Correction: Journal of Nanobiotechnology (2023) 21:339** **https://doi.org/10.1186/s12951-023-02083-y**

The graphic abstract was missing from this article (and should have appeared as below).
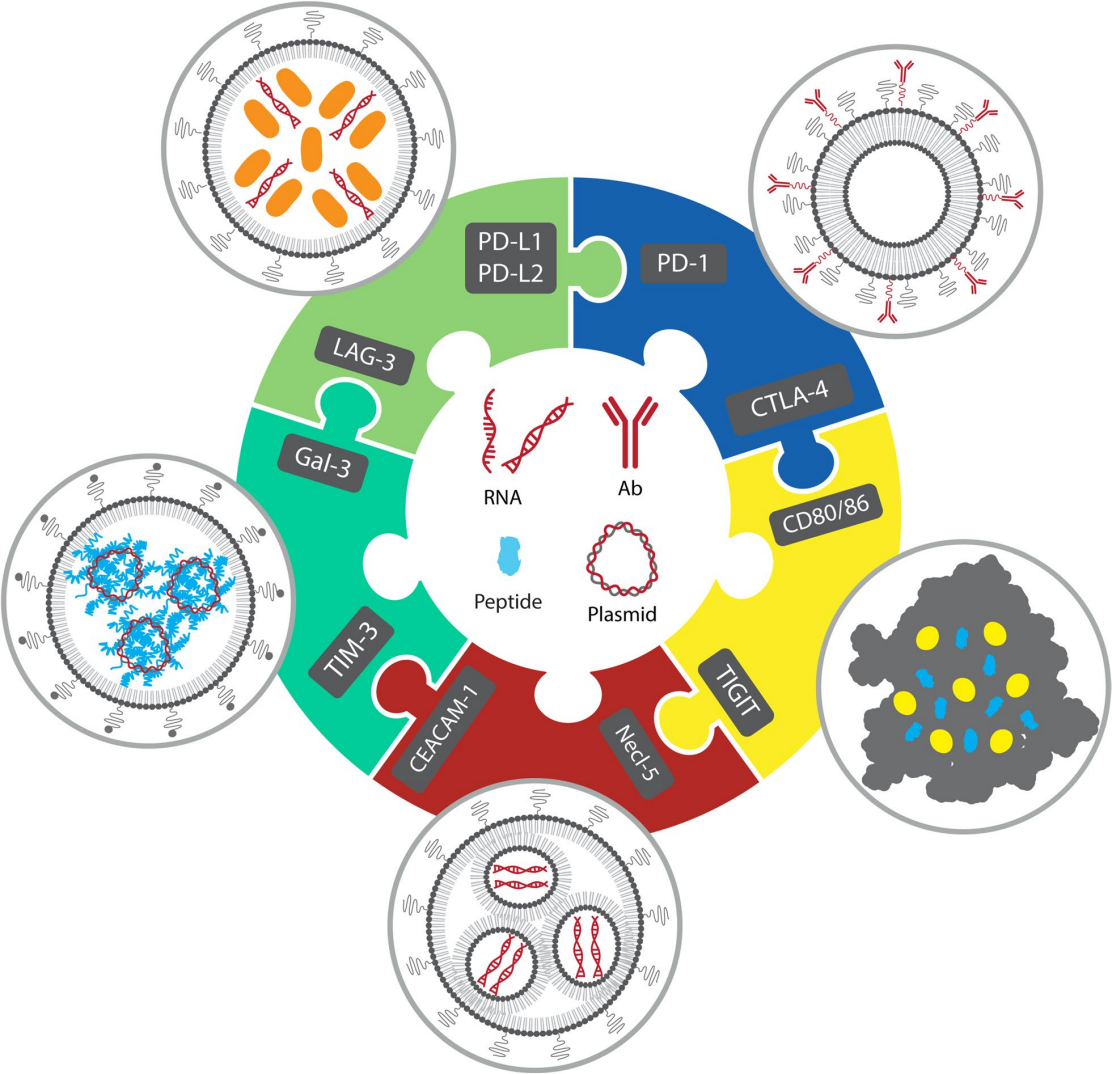


The original article has been revised.
